# Detection of Emerging Pollutants Using Aptamer-Based Biosensors: Recent Advances, Challenges, and Outlook

**DOI:** 10.3390/bios12121078

**Published:** 2022-11-25

**Authors:** Elda A. Flores-Contreras, Reyna Berenice González-González, Everardo González-González, Elda M. Melchor-Martínez, Roberto Parra-Saldívar, Hafiz M. N. Iqbal

**Affiliations:** 1 Tecnologico de Monterrey, School of Engineering and Sciences, Monterrey 64849, Nuevo León, Mexico; 2 Institute of Advanced Materials for Sustainable Manufacturing, Tecnologico de Monterrey, Monterrey 64849, Nuevo León, Mexico; 3 Laboratorio de Fisiología Molecular y Estructural, Facultad de Ciencias Biológicas, Universidad Autónoma de Nuevo León, San Nicolás de los Garza 66455, Nuevo León, Mexico

**Keywords:** biosensors, biosensing constructs, aptamers, bioanalytical measurements, biorecognition, hybrid nanostructures, environmental monitoring, emerging pollutants

## Abstract

The synergistic potentialities of innovative materials that include aptamers have opened new paradigms in biosensing platforms for high-throughput monitoring systems. The available nucleobase functional moieties in aptamers offer exclusive features for bioanalytical sensing applications. In this context, compared to various in-practice biological recognition elements, the utilization of aptamers in detection platforms results in an extensive range of advantages in terms of design flexibility, stability, and sensitivity, among other attributes. Thus, the utilization of aptamers-based biosensing platforms is extensively anticipated to meet unaddressed challenges of various in-practice and standard analytical and sensing techniques. Furthermore, the superior characteristics of aptasensors have led to their applicability in the detection of harmful pollutants present in ever-increasing concentrations in different environmental matrices and water bodies, seeking to achieve simple and real-time monitoring. Considering the above-mentioned critiques and notable functional attributes of aptamers, herein, we reviewed aptamers as a fascinating interface to design, develop, and deploy a new generation of monitoring systems to aid modern bioanalytical sensing applications. Moreover, this review aims to summarize the most recent advances in the development and application of aptasensors for the detection of various emerging pollutants (EPs), e.g., pharmaceutical, and personal care products (PPCPs), endocrine-disrupting chemicals (EDCs), pesticides and other agricultural-related compounds, and toxic heavy elements. In addition, the limitations and current challenges are also reviewed, considering the technical constraints and complexity of the environmental samples.

## 1. Introduction

The increasing agricultural and industrial activities derived from the requirements of a growing population have increased the use of chemicals and environmental pollution by emerging pollutants (EPs). Such emerging contaminants possess a significant risk to human and ecosystem health; they are described as natural or synthetic chemicals or microorganisms that are still not regulated by environmental authorities [1]. Some typical examples of EPs are pharmaceutical and personal care products, endocrine-disrupting chemicals (EDCs), agricultural compounds, and heavy metals. All of them have been detected in environmental samples such as soil samples and water bodies around the globe [2,3,4]. Due to the inability of conventional wastewater treatment plants (WWTPs) to remove this type of pollutants, they are typically released into the aquatic environment by industrial and domestic wastewater [1].

The relevance of mitigating the contamination caused by EPs is based on their characteristics related to bioavailability, mobilization, transformation, and persistence in water, sediments, and soil [5]. Moreover, EPs can be introduced to the food chain through different pathways, such as irrigation with contaminated water and plant uptake [6]. In this manner, researchers have explored the adverse effects of many EPs in organisms. For example, *Pangasius* sp. fish was recently used as a model to analyze the toxic effects of an antibiotic. Several effects were observed in the fish after exposure, including higher levels of antioxidant enzymes, modification of hormones, and physiological changes [7]. Similar studies have demonstrated ill effects on multiple organisms caused by contaminants of emerging concern [8,9].

Therefore, research has been focused on proposing solutions with different approaches, including prevention, remediation, and detection. Some preventive measures involve chemical simplification to reduce the number of chemicals in the manufacturing of consumer products [10]. Studies based on remediation technologies include effective and innovative techniques using nanomaterials and advanced bioremediation techniques [1,11]. On the other hand, research groups have reported the design of novel diagnosis methods to selectively detect some EPs [12,13]. However, the monitoring of such pollutants through highly sensitive, cost-effective, and highly selective technologies is still challenging. Conventional techniques for environmental diagnosis typically require specialized personnel, expensive laboratory facilities, complex sampling or sample preparation, and time-consuming procedures [12,13,14]. Recently, biosensors have been presented as a suitable solution for real-time and on-site monitoring for the detection of multiple pollutants. Relevant advancements have been documented based on the selection of the bioreceptor, which can be formed by cells, enzymes, antibodies, and aptamers, among others. The aptamers are small single-stranded oligonucleotides that present multiple advantages as bioreceptors, such as high selectivity and affinity for specific targets, low cost, short production time, and sensing abilities in inhospitable conditions [12]. In this manner, aptasensors have been employed for the selective detection of different pollutants on different environmental matrices. In this review, the most recent advances in aptamer-based biosensors are discussed to denote their ability to detect pharmaceutical and personal care products, heavy metals, endocrine-disrupting chemicals, and agricultural compounds in different environmental samples. Finally, current challenges faced by aptamer-based technology are presented to provide insight into the possible approaches to solve them.

## 2. Fundamentals of Aptamer-Based Biosensors

### 2.1. Aptamer Development

**Figure 1:**
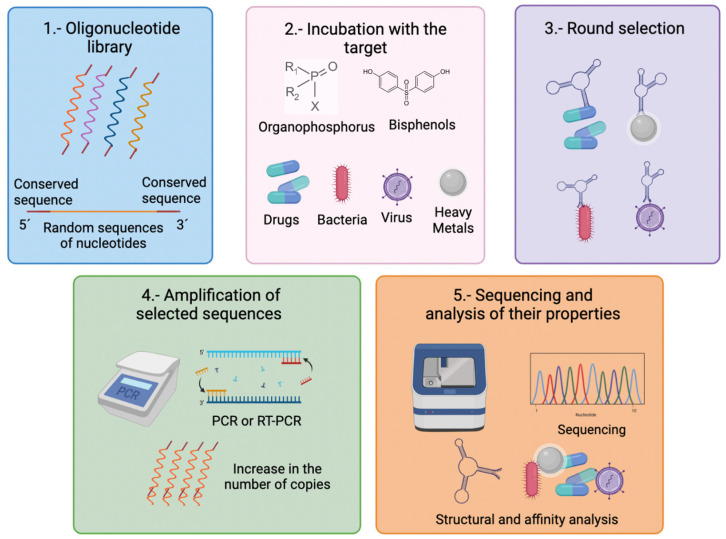
Schematic illustration of the methodology followed for the development of in vitro aptamers by the SELEX method (Systematic evolution of ligands by exponential enrichment). (**1**) Aptamers are formed by DNA or RNA from a library that can be commercial; these sequence at their 5′ and 3′ ends present conserved regions, while their internal nucleotide sequence is random. (**2**) Aptamers are incubated with the target of interest (drugs, viruses, bacteria, endocrine-disrupting chemicals, or heavy metals). (**3**) In total, 8–15 cycles of selection rounds are performed to separate the sequences that present affinity for the target and discard those unbound to the target. (**4**) The selected sequences are amplified through the conserved regions at their 5′ and 3′ ends by hybridizing the primers in these regions. (**5**) Finally, the properties of the selected aptamers are sequenced and analyzed. Created with BioRender.com.

Currently, the technology used for the development of in vitro aptamers is based on the SELEX procedure (Systematic evolution of ligands by exponential enrichment). This process involves the use of an oligonucleotide library of strands of random nucleotide sequences (20–50 nt) that are flanked at their 5′ and 3′ ends by conserved sequences. The sequences obtained from the library are incubated with the analyte of interest. Then, the sequences that bind to their target analyte or molecule are selected and separated from the other sequences. The selected sequences are amplified by PCR (DNA sequences) or reverse transcription PCR (RNA sequences) to obtain a greater number of copies and perform more rounds of selection (8–15 cycles). In the final selection stage, they are sequenced, and some characteristics such as their molecular structure, specificity, affinity, and selectivity towards their target are analyzed (Figure 1) [15,16].

### 2.2. Types of Aptasensors

Aptasensors are devices based on the use of aptamers as the recognition element to detect different analytes of interest. These biosensors have multiple advantages, such as high consistency between batches, low development cost, high chemical stability, sensitivity, non-toxicity to living cells, and ease of modification and synthesis. In addition, they are resistant to changes in pH, temperature, and ionic strength [15,17,18].

**Figure 2:**
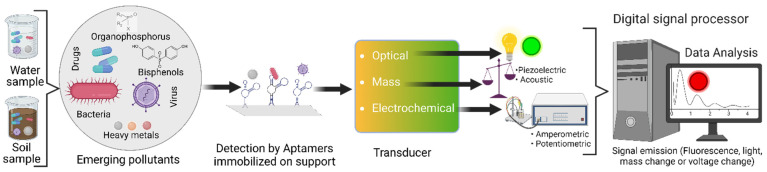
Schematic representation showing the operation of an aptasensor. The soil or water samples are loaded onto the surface of the device, in which the aptamers are immobilized. After the aptamers bind with their target (heavy metals, drugs, bacteria, viruses, or endocrine-disrupting chemicals), they change their conformation and release signals of chemiluminescence, fluorescence, mass change, and changes in electric charge, among others. Created with BioRender.com.

Aptamers can acquire complex three-dimensional (3D) structures, such as G-quadruplexes, multi-branched junctions, or loops, that are immobilized on diverse supports such as electrodes, glass chips, and nano or microparticles through the addition of coatings (streptavidin or avidin), chemical groups (amino, hydroxyl or carboxyl) or terminal modifications (thiol group) [12]. Aptamers bind to their targets by electrostatic and intramolecular interactions (π-π stacking, Van der Waals forces, and hydrogen bonds), recognizing various analytes such as metal ions or molecules as proteins, and even identifying living cells or tissues [17,18]. In addition to the aptamers, aptasensors have a transducer element as well. Transducers can identify the signals released after the interaction of aptamers-targets. These signals can be fluorescence, luminescence, changes in electric charge, potential, mass, and color [19,20]. The different possible signals led to the aptasensor classification in optical, electrochemical, or optical-mass (Figure 2) [20].

#### 2.2.1. Optical Aptasensors

**Figure 3:**
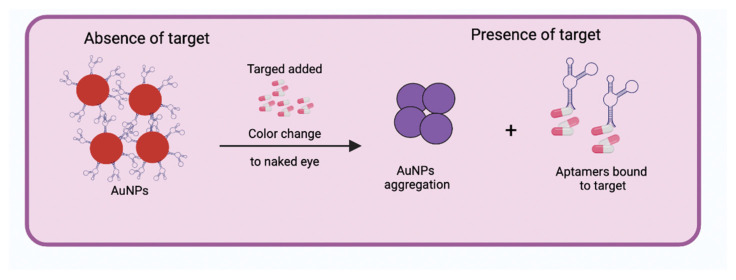
Schematic illustration showing the operation of the colorimetric aptasensor in the absence and presence of its target. In the absence of the target, the aptamers are anchored to the surface of the gold nanoparticles (AuNPs). However, a color change from red to purple is observed with the naked eye when the target is presented, which is caused by the aggregation of the AuNPs after the detachment of the aptamers from the surface of the nanoparticles to be able to bind with their target. Created with BioRender.com.

Typically, optical aptasensors are colorimetric, fluorescence, and chemiluminescence [20]. Colorimetric aptasensors are made up of nanoparticles of noble metals, mainly gold and silver. These types of nanoparticles are known as strong quenchers since they absorb and scatter light. Among the advantages offered by this detection method is the interpretation with the naked eye (presenting a wide range of colors) and real-time monitoring. They also provide high robustness and sensitivity for quantitative measurements, affordable prices, do not require labels, and are easy to handle and transport [15,21]. The design of the colorimetric aptasensors consists of aptamers adhered to the surface of the nanoparticles, which upon binding to their target, change their structural conformation from random coil to folded rigid, which causes the aptamers to be released from the surface of the nanoparticles and these aggregate, leading to a color change to the naked eye (Figure 3) [20].

**Figure 4:**
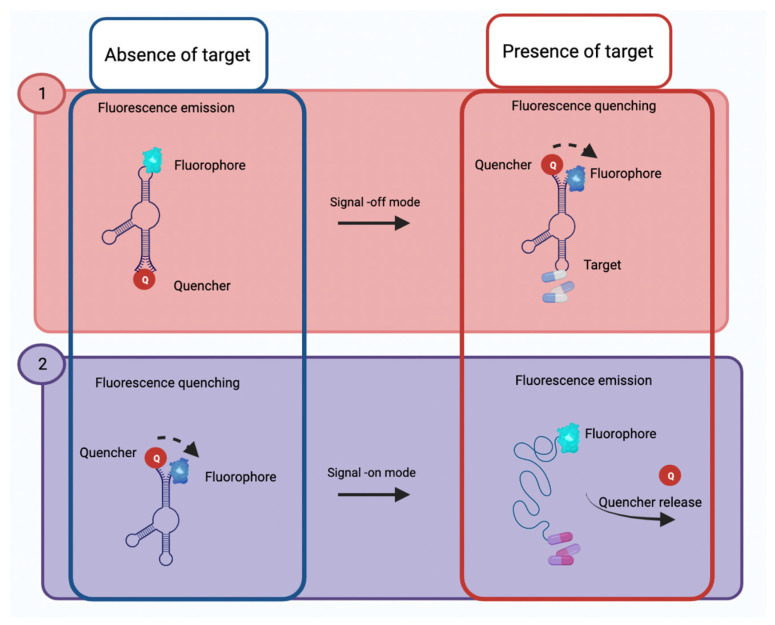
Schematic illustration representing two types of events in fluorescence aptasensors. (**1**) Signal-off mode, in which the aptamers release a fluorescence signal (in the absence of the target) since the fluorophore is far from the quencher; however, when the target is presented, the aptamer undergoes a conformational change, causing the fluorophore to approach the quencher and turn off the fluorescence signal. (**2**) Signal-on mode, in which the fluorophore is close to the quencher extinguishing the fluorescence signal (in the absence of the target), but if the target is presented, a conformational change occurs, releasing the quencher to bind to its target. Created with Biorender.com.

Regarding fluorescent aptasensors, aptamers are commonly labeled with a fluorophore and a quencher, using the Förster resonance energy transfer (FRET) mechanism, which consists of the transfer of energy from a donor (excited chromophore) to an acceptor (second chromophore), through non-radiation (dipole–dipole). In line with this, the labeled aptamer with a fluorophore—in the presence of its target—changes its conformation, which can give rise to two types of events (Figure 4), the first being that the fluorophore is close to the quencher extinguishing the fluorescence (signal-off mode) or the second event; that the conformational change causes the fluorophore to move away from the quencher generating a fluorescence signal (signal-on mode), these types of events will depend on the design of the aptasensor. Different fluorescence emission elements have been used: fluorescent probes (FAM, CY3, or CY5) or fluorescent nanomaterials such as upconversion nanoparticles (UCNPs), quantum dots (QDs), silver nanoclusters (AgNCs)-DNA, silver nanoparticles (AgNPs)-EU (III), or quenchers such as graphene oxide (GO) and organic quenching molecules [22]. These fluorescence-based aptasensors are low in cost, and the fluorescence signal correlates with the concentration of the analyte [15,20,23].

**Figure 5:**
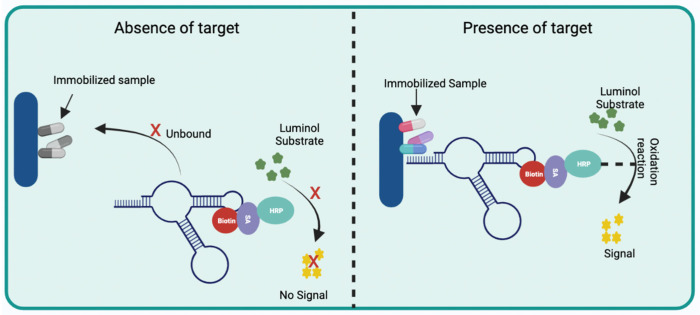
Schematic representation showing the operation of a chemiluminescent aptasensor. This aptasensor consists of an immobilized sample, which does not interact with the biotin-labeled aptamer when the target is not presented. Streptavidin (SA) conjugated with horseradish peroxidase (HRP) is discarded after washing; therefore, in the presence of luminol, an oxidation reaction does not occur, avoiding a chemiluminescent signal. On the other hand, when the target is presented, the biotin-tagged aptamer is immobilized, which then interacts with the HRP-conjugated SA, releasing a chemiluminescent signal in the presence of its substrate. Created with BioRender.com.

Unlike fluorescence-based transducers, chemiluminescent sensors do not need external stimuli of light sources. Their performance involves an oxidation reaction that releases energy and excites certain molecules, causing the emission of light. Chemiluminescent aptasensors commonly consist of a biotin-labeled aptamer, which, when interacting with its target, allows streptavidin (SA)-conjugated peroxidase (HRP) to bind with biotin and, in the presence of luminol release a chemiluminescent signal (Figure 5) [20,23,24].

#### 2.2.2. Electrochemical Aptasensors

**Figure 6:**
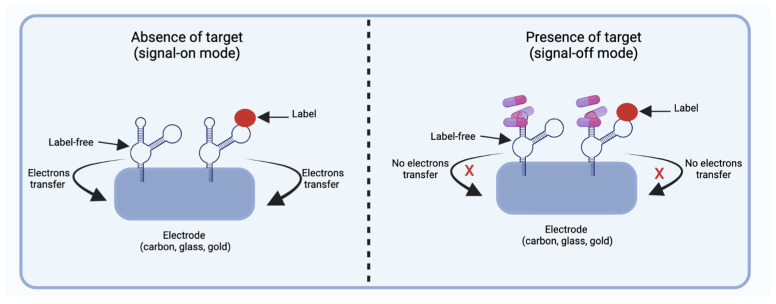
Schematic representation showing the operation of an electrochemical aptasensor in two different events (signal-on mode and signal-off mode). Signal-on mode occurs in the absence of the target; labeled and unlabeled aptamers are immobilized on the electrode surface, promoting electron transfer. Contrarily, in signal-off mode, the immobilized aptamers (labeled and label-free) interact with the target causing the inhibition of electron transfer. Created with BioRender.com.

Electrochemical aptasensors are formed by an electrode (e.g., carbon, glass, gold) on the surface of which aptamers are immobilized and can be labeled or label-free. Labeled aptamers are covalently or non-covalently (interactions electrostatically) bound to their labels, such as redox compounds, probes (e.g., iron oxide, methylene blue, or ferrocene), metal nanoparticles, antibodies, and enzymes. In comparison, label-free aptasensors consist of a sensing electrode that is affected by variations in electrical charge. Therefore, electrochemical aptasensors with labeled or label-free aptamers are sensitive to electron transfer. In the absence of their target, the labeled and free labels aptamers are found near the electrode, promoting the transfer of electrons, and releasing an electrochemical signal defined as signal-on mode (Figure 6). While in the presence of its target, the aptamer undergoes a conformational change, inducing labeled and label-free aptamers to move away from the electrode surface, causing mode signal-off (Figure 6). This type of design of signal-on mode and signal-off mode may vary depending on the manufacturer. Electrochemical aptasensors are mainly classified into four groups: conductometric, amperometric/voltametric, impedimetric, and potentiometric [20]. Conductometric aptasensors detect charge change by binding the aptamers to their target, while the amperometric/voltometric aptasensors indicate the differences in the current of potentials after the aptamers undergo a conformational change in the presence of their target [20]. Impedance aptasensors are based on the measurement of the opposition of the flow of electrons in an alternating current circuit. When aptamers undergo a conformational change in the presence of their target, it leads to inhibition of charge transfer (aptamers move away from the electrode), inducing a high impedance. Contrary to when the aptamer is free, causing charge transfer (aptamers are close to the electrode) and a low impedance [1,2], while potentiometric aptasensors measure the potential difference in the presence of their target, which causes the aptamers to undergo a conformational change, inducing charge variations from negative to positive [1].

#### 2.2.3. Optics-Mass Aptasensors

**Figure 7:**
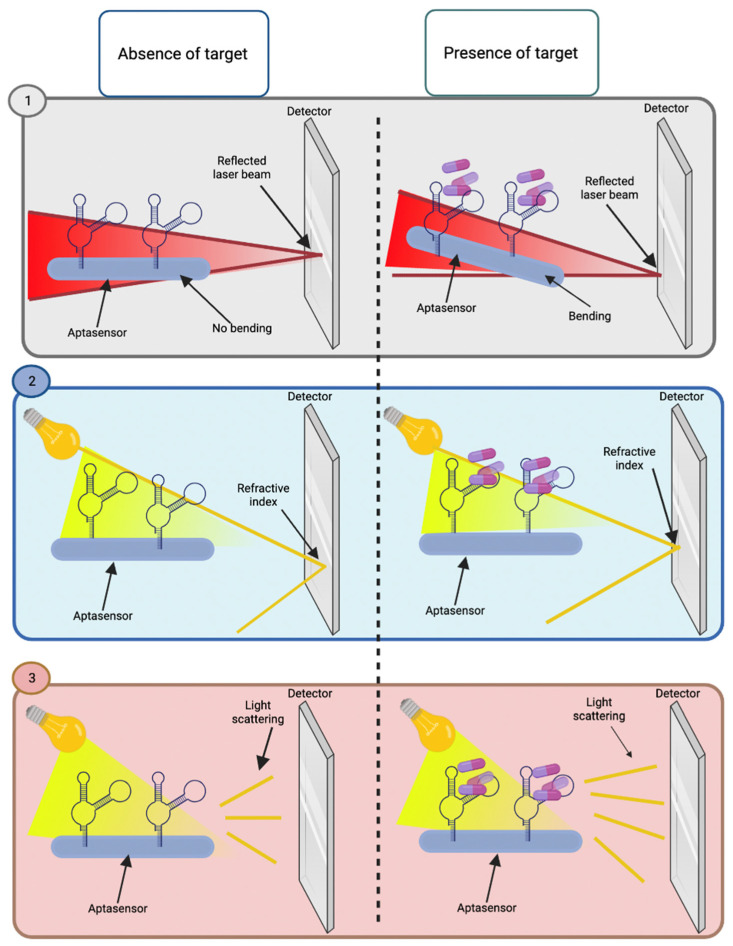
Schematic representation of the different types of optical-mass aptasensors and their operation in the absence and presence of the target. (**1**) The cantilever array aptasensor consists of aptamers immobilized on its surface; in the absence of the target, no bending or change in the laser beam reflected is detected. In contrast, when the target is presented, the detector identifies bending and a change in the position of the reflected laser beam. (**2**) Surface plasmon resonance (SPR) aptasensor consists of immobilized aptamers; in the presence of the target, a change in the refractive index is identified by the detector, which is not detected in the absence of the target. (**3**) Surface-enhanced Raman scattering (SERS) consists of aptamers immobilized on the surface of the aptasensor, which detects light scattering after the input of incident light. No changes are observed in the absence of the target, contrary to when the aptamers interact with their targets. Created with BioRender.com.

Other widely used aptasensors are optical-mass sensors, which are very sensitive, do not need labels, and provide results in short analysis periods. These are based on the mass change detected on the surface of the aptasensor, generating an optical signal. The most common types of transducers for optical-mass aptasensors are the cantilever array, surface plasmon resonance (SPR), and surface-enhanced Raman scattering (SERS) (Figure 7). Cantilever array aptasensors detect optical signals that are generated by the change in mass caused by the binding of the aptamer to its target. The aptamers are immobilized on the surface of the self-assembled monolayers, which after binding to their targets, would make the cantilever bend. This bending is measured to determine the position of the reflected laser beam in the detector [23]. Regarding SPR, it is based on the change in the refractive index caused by the binding of aptamers to their targets, which are immobilized on the metal surface of the aptasensor. While SERS-based aptasensors are formed by aptamers immobilized on the metal surface (Au or Ag), when aptamers bind with their targets, the input of incident light interacts with the sample, causing light scattering. [15,20,23]. Another type of aptasensors is the quartz crystal microbalance (QCM), which is an acoustic type of sensor that consists of a quartz disk with electrodes. The aptamers are immobilized on the surface of the aptasensor, which, when interacting with their target, would generate a change in the frequency of resonance [2,3]. Various combinations of transducers have also been reported, such as optical-electrical, electrical-mass, and electro-chemiluminescent, among others, which are based on the basic principles of the binding of aptamers with their targets, and the generation of a signal that allows the qualitative or quantitative identification of the presence of an analyte of interest [23].

## 3. Recent Advances in Aptamer-Based Biosensors

In recent times, aptamers have gained significant research attention as a recognition element in the development of biosensors for multiple applications. These oligonucleotide sequences or their modifications can detect biological contaminants, pharmaceutical pollutants, heavy metals, and endocrine-disrupting chemicals, among other EPs (Table 1).

### 3.1. Biological Contaminants

The presence of biological contaminants in water, soil, and food matrices represents a serious global concern for public health. Viruses and pathogenic bacteria are some examples belonging to this type of pollutants, which are associated with negative effects in humans, such as fever and diarrhea. The concern for the hazards related to biological contaminants has led the U.S. Environmental Protection Agency to report 12 microbial contaminants, including bacteria, protozoa, and viruses, in their draft fifth contaminant candidate list (CCL5) [43]. The presence of different biological contaminants has been reported around the world in drinking water, surface water, and water treatment plants, among other matrices [44,45,46]. Therefore, research efforts have been focused not only on the development of removal techniques but in innovative techniques for accurate detection [47,48]. For instance, Zheng et al. [25] created a portable photoelectrochemical aptasensor in order to detect *E. coli* present in water samples. Their aptasensor was based on electrochemical impedance spectroscopy and consisted of indium-tin-oxide electrodes covered with cadmium sulfide quantum dots and gold nanoparticles (AuNPs) to which the aptamers bind. Through photocurrent changes, the aptasensor was able to identify *E. coli* after approximately 40 min; the linear range was reported from 4 × 10^2^ CFU/mL to 4 × 10^7^ CFU/mL, with a limit of detection (LOD) of 200 CFU/mL, showing high selectivity and reproducibility [25]. *Salmonella typhimurium* is another bacterium of great environmental concern; thus, Ren et al. [26] created an aptasensor for its detection in water or food samples using the principle of fluorescence and based on a magnetic separation system.

The simultaneous detection of different strains or species is still challenging; however, recently, Fang et al. [49] reported the development of a novel biosensor able to detect *E. coli* and *S. typhimurium* simultaneously. The biosensor was developed using two different aptamers: Cy3-apt-E and Cy5.5-apt-S, which are specific for the detection of *E. coli* O157:H7 and *S. typhimurium*, respectively. In their system, free aptamers (not bound to bacteria) can enter the nanopore layer of the nanoprobe and emit greater fluorescence when are excited in comparison to aptamers bound to bacteria. A decrease in fluorescence signal is resulted since the complex formed between bacteria and aptamers prevents the entrance to the nanopores [49].

Similarly, an aptasensor developed by Lu et al. [27] can detect multiple foodborne bacteria, such as *S. typhimurium*, *E. coli* O157:H7, and *S. aureus* after approximately 10 min of reaction, being useful for the monitoring of environmental samples. This biosensor is a lateral flow test strip based on the aptameric sandwich model, and it consists of a sample pad and a pad of conjugates where the primary aptamer acts as a signal probe conjugated to AuNPs. Subsequently, a second aptamer is immobilized in the test line, which captures the targets-Aptamer 1-AuNPs on nitrocellulose membrane, generating a red line in the lateral flow assay in the presence of bacteria. Contrarily, in the absence of bacteria, the complex Aptamer 1-AuNPs migrates to the control pad, and a red line is observed. In this manner, the presence of bacteria will be indicated by two red lines in the lateral flow assay. The LOD for this biosensor has been reported as 103 CFU/mL, 104 CFU/mL, and 104 CFU/mL for *S. typhimurium*, *E. coli* O157:H7, and *S. aureus*, respectively. It should be noted that this lateral flow aptasensor is not able to identify the individual presence of each bacterium [27].

On the other hand, Peinetti et al. [28] developed an aptamer-nanopore sensor capable of detecting SARS-CoV-2 and human adenovirus. Their sensor consisted of single nanochannel membranes of polyethylene terephthalate (PET); aptamers were immobilized on the inner wall of the nanopore, and the presence of SARS-CoV-2 and human adenovirus was identified due to variations in conductance. The linear range was reported from 1 × 10^4^ to 1 × 10^8^ copies/mL for SARS-CoV-2 and from 6 pfu/mL to 6 × 10^4^ pfu/mL for human adenovirus, whereas the LOD was reported of 1 × 10^4^ copies/mL and 1 pfu/mL, respectively. The aptasensor required around 30 min to provide results of water, saliva, and serum samples.

### 3.2. Pharmaceutical and Personal Care Products

Pharmaceuticals and personal care products are a source of EPs that have received increased attention in recent times. Their adverse effects on aquatic organisms and humans can be presented even at low concentration levels. Of main relevance is the antimicrobial resistance that has been exacerbated by the inadequate treatment or disposal of antibiotics, which is considered a serious health problem of major concern by the World Health Organization [50]. The increasing consumption of pharmaceutical products, along with the high tendency to dispose of them inadequately, either with regular garbage or flushed into the sewer system through the sink or toilet, have led to its presence in environmental samples [51]. Rivers, lakes, and aquatic organisms have exhibited high concentrations of pharmaceutical pollutants leading to negative effects [52,53]. In this manner, it is imperative the development of simple, inexpensive, and sensitive biosensors for the monitoring of such pollutants in environmental matrices.

**Figure 8:**
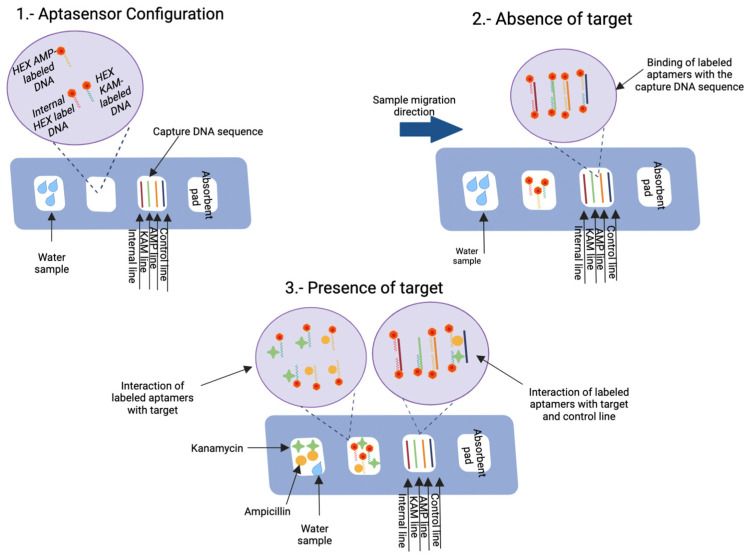
Schematic representation showing the setup and operation of an aptamer-based rapid test for the simultaneous detection of ampicillin (AMP) and Kanamycin (KAM). (**1**) The aptamer-based rapid test consists of four sections: in the first section, the sample is loaded; in the second section, the labeled aptamers that recognize the positive control (internal line) are located; the third section contains DNA sequences immobilized with biotin to capture the labeled aptamers; the fourth section consists of an absorbent pad. (**2**) In the absence of the target, the loaded sample in the first section migrates by capillarity to the second section, in which the labeled aptamers are located; they move to the third section, where the DNA sequences capture their corresponding aptamers. The internal line traps the internal HEX label DNA, the KAM line captures HEX KAM-labeled DNA, the AMP line captures HEX AMP-labeled DNA, and the control line captures some of the remnants of the HEX KAM-labeled DNA, and HEX AMP-labeled DNA aptamers. (**3**) In the presence of the target, the sample migrates from section one by capillarity to the second section where the KAM and AMP targets bind to their HEX-AMP-labeled DNA and HEX AMP-labeled DNA aptamers, respectively. In section three, these target-bound aptamers are captured in the control line, while free aptamers are bound in the AMP and KAM lines. Therefore, the control line signal is inversely proportional to the AMP and KAM lines. Created with BioRender.com.

Different aptamer-based sensors have been developed for this purpose. For instance, Lin et al. [29] presented a rapid lateral flow assay able to simultaneously detect ampicillin (AMP) and kanamycin (KAM) in water samples after 10 min of reaction. The aptasensor worked in concentrations of AMP and KAN ranging from 0.50 to 500 ng/L and from 0.1 to 1000 ng/L, respectively, with a LOD for AMP of 0.06 and for KAM of 0.015 ng/L. The lateral flow strip consisted of four sections (Figure 8). In the first section, the samples are placed and migrated by capillarity. The second section, or the conjugate pad, consists of different aptamers, including an internal HEX label DNA (control), HEX KAM-labeled DNA, and HEXAMP-labeled DNA, that recognize KAM and AMP, respectively. These aptamers are labeled with fluorophore HEX (Hexachloro-6-carboxyfluorescein), which is responsible for recognizing their targets if they are presented in the sample. Then, they migrate towards the third section or the test line, in which oligonucleotides are immobilized on the nitrocellulose membrane. The first line is the oligonucleotides that recognize the aptamer Internal HEX-labeled DNA; the second and third line is the oligonucleotides that captured the free KAM and AMP aptamers, respectively; and the fourth line is the oligonucleotides that captured the aptamers bind to KAM and AMP. Therefore, in the presence of AMP and KAM, four lines are generated in the test section [29].

Similarly, Xu et al. [30] developed an aptasensor capable of detecting two antibiotics simultaneously in approximately thirty minutes of reaction. It consisted of magnetic beads attached to capture probes complementary to aptamers that recognize oxytetracycline (OTC) and KAM. The aptamers—in the presence of OTC and KAM—are released from the capture probes due to their high affinity towards their targets. The free probes capture the AuNPs modified by HRP (horseradish peroxidase) that catalytically oxide TMB (3,3′,5,5′-tetramethylbenzidine) and O-phenylenediamine (OPD), generating a color change that is proportional to the concentration of OTC (at 370 nm) and KAM (at 450 nm). This aptasensor has a low LOD of 1 ag/mL for both OTC and KAM; antibiotics could be quantitatively detected in an ample range from 10^−^^6^ to 10^5^ pg/mL [30].

### 3.3. Endocrine-Disrupting Chemicals

Endocrine-disrupting chemicals (EDCs) such as phthalates and bisphenols are contained in commercial products typically used by consumers. Their presence in environmental samples represents a serious concern due to their high persistence, toxicity, and bioaccumulation. EDCs can alter the natural functions of hormones causing negative effects in humans and animals even at low concentrations [31,33]. The adverse effects associated with EDCs on the growth and development of aquatic organisms, wildlife, and humans have been widely studied by the research community. It has been documented evidence of bioaccumulation and alteration of different systems, including the endocrine and immune [1,54]. Thus, the development of biosensing tools for the opportune detection of this type of pollutant is highly relevant.

Diverse aptasensors have been developed for the detection of endocrine-disrupting chemicals. For instance, Tu et al. developed an aptasensor to determine the presence of phthalate. The SERS-based aptasensor consisted of aptamers immobilized on magnetic particles that have an affinity for Bis(2-ethylhexyl) phthalate (DEHP). In addition, this system has a Raman reporter molecule conjugated to the silver nanoclusters that present the DEHP analog anchored, which competes for the aptamers in the presence of DEHP. This aptasensor presented a LOD of 8 pM and a detection range of 0.008–182 nM [31]. Similarly, Chen et al. developed a colorimetric aptasensor able to detect phthalate; however, they used gold nanoparticles wrapped by truncated aptamers instead of full-length aptamers (selected by selex). These truncated aptamers, the mers that are not effective for binding with their target, are eliminated, which allows recognition of multiple structures such as phthalate esters. This aptasensor has a LOD of 1 ng/L and a detection range of 0.003–10 µg/L [32].

Regarding aptasensors to detect bisphenols, Wei et al. created a fluorescent aptasensor that indicates the presence of bisphenol A in tap water. This system was formed by the fluorescence messenger berberine, which embeds itself in the random coil of the aptamer that recognizes bisphenol A. In the absence of its target, a fluorescence signal is emitted, whereas, in the presence of bisphenol A, the aptamer undergoes a conformational change forming a hairpin structure and releasing the berberine, quenching the fluorescence signal. This sensor has a LOD of 32 nM and recognizes a concentration range of 0–1300 µM [33]. Additionally, Li et al. developed an aptasensor that detects bisphenol A but uses a SERS transducer. This aptasensor is constructed with a thiolate aptamer probe specific to bisphenol A. This aptamer is bound to 4-mercaptobenzoicacid (4-MBA)-embedded gold/silver core-shell nanostructures that are complementary to the DNA sequence anchored in CoFe_2_O_4_@HNTs -AuNPs nanocomposites. In the presence of bisphenol A, aptamers dissociate from the complementary DNA sequence, causing a negative correlation with bisphenol A concentration and Raman intensity. This aptasensor has a LOD of 0.75 pg/mL [34].

### 3.4. Heavy Metals

Heavy metal contamination is a global environmental problem of great concern due to its alarmingly toxic levels, persistence, and nonbiodegrability. Different sources of metal pollution have been identified, including natural and anthropogenic sources. Rapid industrialization has exacerbated the situation; activities such as mining, packaging, plastic manufacture, and coal combustion have been reported as main contributors to the extensive discharge of metal ions into environmental matrices [55,56]. Metals such as Cr, As, Cd, and Cu, among others, have been detected in surface water, groundwater, and sediments around the world [57,58]. Moreover, they can accumulate for long periods in the environment and ascend through the food chain. In this regard, researchers have demonstrated the negative effects of metal pollution on the health of aquatic organisms [59]. Such consequences are also observable in human health after the accumulation of toxic metals at certain concentrations; growth, development, metabolic, and neuromuscular disorders have been associated with metal toxicity [60]. Various techniques have been applied for the detection of metals, including optical-based, electrochemical-based, and spectroscopic-based techniques; however, several drawbacks still need to be overcome, such as geographic limitations of contaminated locations, high costs, and requirement of qualified personnel, among others [61]. In this manner, the research community has explored innovative developments for the sensitive and selective detection of heavy metal ions [62,63].

The applications of aptamer-based biosensors have been extended to the detection of metals. For instance, Ding et al. constructed an electrochemical aptasensor capable of detecting Pb^2+^ in a concentration range of 0.1–10 µg/L with a low LOD of 0.096 µg/L in both polluted water and soil samples. The designed aptasensor consisted of a screen-printed carbon electrode and a carbon material derived from core-shell zeolite imidazole frameworks to which the aptamers were immobilized. The aptamers were able to recognize Pb^2+^ and measure the cyclic voltammetry. Furthermore, this aptasensor exhibited reproducibility and practicability; however, high concentrations of Ag^+^, K^+,^ and NH^4+^ caused interferences in the performances suggesting that further research should be performed in order to avoid such effects [35].

Another type of aptasensor developed for the detection of Pb^2+^ in polluted water was presented by Li et al. [36]. The aptasensor was prepared using nitrogen-doped carbon quantum dots (NCQDs) possessing carboxyl groups which were placed on a glassy carbon electrode. Subsequently, aptamers were added to NCQDs to obtain a stable aptasensor. NCQDs were selected as the electrochemiluminescence material due to their unique properties, including water solubility, high fluorescence, ease of preparation, low manufacturing cost, exceptional electrical and optical properties, and ease of surface functionalization. NCQDs were doped with N element to enhance the signal. Regarding the operation and like other aptasensors, its functionality was based on the aptamer that interacts with Pb^2+^ causing modification on its conformation from a single strand to G-quadruplex. This modification results in a fluorescence decrease since Pb^2+^ acts as a quencher, blocking the transfer of electrons between the NCQDs and co-reactant peroxydisulfate. This biosensor worked on a detection range from 50 pM to 387.9 nM with a LOD of 18.9 pM; in addition, its fabrication was considered low-cost and environmentally friendly [36].

Different aptasensors have been developed for the detection of arsenic (As^3+^) in environmental samples. For example, Sidduqui et al. [37] reported a method for the detection of As^3+^ in polluted soil samples. The method involved sample preparation and smartphone-based optical detection. Simplification and optimization of sample preparation were achieved by acid extraction and the serial application of different phases of soil extraction to remove interfering ions. Then, As^3+^ detection was performed by a colorimetric method using an aptamer (Ars-3) and gold nanoparticles (AuNPs). The Ars-3 is strongly bound to AuNPs due to their respective surface charges; however, when As^3+^ is present, the metal ions are absorbed on the surface of AuNPs, inhibiting the stable binding of Ars-3-AuNPs, which in turn causes the NaCl-induced aggregation and the color change from red to purple. The colorimetric change is detected by a smartphone, and it allows the sensitive detection of As^3+^ in soil and aqueous samples with low LODs of 1.97 ppm and 14.44 ppb, respectively. Similar to Sidduqui et al. [37], other researchers have reported the use of Ars-3 aptamers to detect As^3+^; however, Zong et al. recently reported that Ars-3 aptamer does not bind specifically to As^3+^. In their work is suggested that the detection of As^3+^ might be associated with adsorption processes that occur between the metal ions and the surfaces of AuNPs, which are typically used in such aptasensors [64]. Despite these findings, biosensors formed by this aptamer have shown high selectivity to determine As^3+^.

On the other hand, Pan et al. [38] determined the concentration of As^3+^ in different water samples using a fluorescence-based biosensor. The biosensor consisted of a triple-helix molecular switch that included a label-free signal probe as a transducer (STP) and a target-specific hairpin central sequence (C-C mismatch of the central sequence). The aptamer has two protruding segments of arms (3′ and 5′ ends) that recognize the STP, 2-amino-5,6,7-trimethyl-1,8-naphthyridine (ATMND) fluorescent molecules that bind to the C-C mismatch of the central sequence, and Exonuclease III (Exo III). In the absence of As^3+^, STP and ATMND bind to the central region of the aptamer, causing low fluorescence and the inability of Exo III to cut the protruding ends of the aptamer. Contrarily, in the case of polluted samples, As^3+^ binds the central region of the aptamer, releasing ATMND and STP. Then, STP binds to the arms of the aptamer, causing Exo III to cut the protruding ends, and STP is released and recycled (can rejoin to C-C mismatch of the aptamer in the absence of As^3+^). Therefore, the observed fluorescence is directly proportional to the As^3+^ concentration. The authors reported a LOD of 5 ng/L for this aptasensor and a detection range from 10 ng/L to 10 mg/L in water samples.

Different aptasensors have reported good performances in the detection of As^3+^ in aqueous samples. Ensafi et al. developed an electrochemical aptasensor composed of a glassy carbon electrode covered by a three-dimensional layer of reduced graphene oxide-modified/gold nanoparticles. Thiolate aptamers were introduced in the system to covalently interact with AuNPs. The presence of As^3+^ caused an increase in the value of the charge transfer resistance on the receptor layer. This aptasensor exhibited a LOD of 1.4 × 10^−^^7^ ng/mL with a detection range from 3.8 × 10^−^^7^–3.0 × 10^−^^4^ ng/mL [39].

Another metal that requires the development of innovative sensors for its detection is cadmium. Xue et al. [64] fabricated an aptasensor based on the dual-polarization of interferometry; it was capable of detecting cadmium II (Cd^2+^) in real-time with a LOD of 0.6 µg/mL. The biosensor presented a layer-by-layer assembly, with a bare silicon oxynitride surface covered with poly-ethyleneimine (PEI) polymer, providing a positive charge on the chip surface. In this manner, T30 oligonucleotides were immobilized through electrostatic interactions, which in turn acted as immobilizers for the aptamers that recognized Cd^2+^ ions. The aptamers presented modification in their conformations depending on the concentration of Cd^2+^. For example, at low Cd^2+^ concentrations, Cd^2+^ ions interact with phosphate groups, causing the single strand of aptamer DNA to stretch and present some vertical hairpin structures. On the other hand, the single strand of the aptamer with few hairpins becomes a narrow and short hairpin structure when interacting with high Cd^2+^ concentrations. These modifications in the conformation of aptamers are detected by dual-polarization of interferometry.

Zhu et al. developed a fluorescence-based aptasensor in which the fluorescence signal is indirectly proportional to the Cd^2+^ concentration. The principle of this biosensor relies on an aptamer that acts as a multifunctional probe. The conformation of the aptamer is changed from a random coil sequence to a stem-loop structure in the presence of cadmium-containing samples. The conformation changes caused the 5′-end labeled with the G4 quencher to get closer to the 3′-end labeled with a 6-FAM fluorophore, thus, decreasing the fluorescence signal. Contaminated water samples were tested, and the biosensor exhibited a LOD of 2.15 nM [40].

### 3.5. Agricultural Compounds

Pesticides, hormones, plant-protection chemicals, and fertilizers are agricultural compounds employed to control diseases and pests and are therefore intended to increase agricultural productivity. They have been used in excessive amounts to maximize productivity, trying to meet the high demands of a growing population [1]. However, their utilization has great implications for agrochemical pollution and the occurrence of such pollutants in environmental samples. In this manner, recent studies have documented the presence of many agrochemical compounds, such as pesticides, in rivers, lakes, sediments, and soil samples [3,65,66]. The monitoring and mitigation of these pollutants are crucial since they can unbalance ecosystems and cause multiple adverse effects on non-target organisms.

Pesticides have been successfully detected with biosensors using aptamers as the recognition component. For instance, Seop et al. [41] developed a colorimetric-based aptasensor capable of identifying iprobenfos (IBF) and edifenphos (EDI) between a concentration range from 10 nM to 5 nM in rice samples. This aptasensor is composed of aptamers that are bound to AuNPs. When the target is not presented in the analyzed samples, the aptamers do not present aggregation in the presence of sodium chloride. Contrarily, the analysis of samples containing IBF and EDI results in a color change from red to purple due to the aggregation of AuNPs, since the aptamers are absorbed by their targets. The aptasensor allows a rapid determination since the results can be obtained after an estimated detection time of 10 min, and color signal can be observed with the naked eye.

On the other hand, Zhang et al. [42] prepared an aptasensor for the detection of several pesticides (phorate, profenophos, and isocarbophos) using fluorescence signals. During the analysis of non-contaminated samples, the aptamers were attached to a molecular beacon (MB), emitting a fluorescence signal, whereas, in the presence of target pesticides, the fluorescence signal decreased. The quenching effect is caused by the greater affinity of the aptamers for their targets, which in turn releases the MB probe and modifies the linear structure (MB bind to aptamer) to hairpin structure. In this manner, the fluorescence emitted by the FAM fluorophore presented at the 5′ end is quenched due to the closeness to the DABCYL quencher present at the 3′ end. Real-time monitoring was possible with this aptamer, showing LODs of 19.2 nM, 13.4 nM, 17.2 nM, and 23.4 nM for phorate, profenophos, isocarbophos, and omethoate, respectively.

## 4. Current Challenges and Recommendations

Nowadays, the requirements to monitor emergent pollutants are increasing derived of their harmful effects on human and animal life. A lot of efforts in researching and developing aptamer-based biosensors to detect toxic targets have been reported with various signal outputs. However, there are several drawbacks to consider in the implementation on-site. For example, the affinity of the aptamers for their targets can be affected, decreasing sensitivity and specificity, causing false positives or negatives, depending on the complexity of the matrix, or using aptasensors that recognize multiple analytes. The high affinity of aptamers for their target correlates with their detection at low concentrations [4,5].

Therefore, chemical modifications can be made to increase the affinity by adding new nucleobases, such as 7-(2-thienyl) imidazo [4,5-b]pyridine (Ds), which promotes the affinity by up to 100 times. Additionally, the affinity can be increased through modifications in the 5-position of the nucleotide deoxyiridin triphosphate (dUTP) by adding aromatic or aliphatic functional groups linked by amide bonds. In addition, artificial DNA analogs are also used to stimulate the affinity of aptamers for their target, such as nucleic acid peptides, instead of ribose phosphates. Another alternative to increase the affinity is through aptamers that present cooperative binding interactions. They are capable of simultaneously binding to different sites on the same target, which is achieved by using multiple aptamers that are ligated onto one strand of DNA [6]. The affinity of aptamers for their target can also be promoted under native conditions through the use of SELEX variants such as in vivo-SELEX or Cell-Selex. These types of protocols allow the selection of aptamers under real physiological or environmental conditions [6,7].

Other possible limitations are associated with the commercialization stage of this type of biosensors. For example, it has been reported the degradation of nucleic acids by nucleases in real environments. New strategies to improve the stability of the aptamers through modified nucleotides, by including chemical groups (2′-*O*-methoxy or 2′-fluoro or motifs) to replace 2′OH moieties, or by the addition of hairpin structures to the 5′ or 3′ ends have been proposed [5,6]. In addition, the backbone of the aptamers, which are phosphodiester bonds, can be substituted by residues of phosphorothioate and boranophosphate or nucleic acid peptides [6].

A very important aspect is the type of aptasensor; for example, optical aptasensors have the advantage of providing results quickly to the naked eye or easy-to-handle instruments; however, optical aptasensors based on color change, such as AuNPs, have the great limitations of requiring very specific conditions such as certain pH values and high concentrations of NaCl (aggregation inducer). Acidic pH values allow aptamers to bind stably to the surface of citrate-coated anionic AuNPs. In the presence of their target, aptamers detach from the AuNPs, with the possibility that other molecules of no interest bind to the surface of the nanoparticles, causing them not to aggregate in NaCl, resulting in false negatives [6,8]. To overcome this obstacle, Qi et al. proposed the use of cationic AuNPs modified with amines, which do not require salt to change color in the presence of the target of interest [9].

Furthermore, optical aptasensors present the great obstacle of not being able to be re-used since aptamers are immobilized on nanoparticles, or they are labeled with a fluorophore or an enzyme, which are immersed in solution (buffer), and the presence of the target is discarded [10]. In contrast, electrochemical or optical-mass aptasensors are usually immobilized on a surface (electrode) and can detect very low concentrations of the analyte (ppm). However, they require expensive materials and a complex manufacturing process [11]. Thus, it is necessary to propose more novel, sustainable, and economical nanomaterials that can be reusable either from a synthetic or natural origin. In addition, a better understanding of their functioning and physical properties is required [12]. Currently, to improve some properties of aptasensors such as detection limits, reproducibility, and sensitivity, research groups are exploring the use of nucleic acid-based nanostructures such as DNA nano strain, DNA nanotetrahedron, and DNA origami since they are compatible with DNA or RNA aptamers, creating supramolecular structures with certain shapes and size [11,13].

Regarding reusable aptasensors, lab-on-a-chip (LOC) devices have been designed which integrate aptamer-based sensors allowing automated washing since the binding between the aptamer and the target is reversible. These devices are portable and low-cost; however, there are still some limitations to overcome, such as the low affinity of the aptamers to their analytes due to the sample matrix. In addition, non-specific interactions between the sample and the aptamers might be presented in multiplex aptasensors, causing false positives [14,15]. Therefore, it is important to increase the affinity of the aptasensors by chemical modifications in the nucleobases, adding artificial DNA analogs, developing aptamers with cooperative binding interactions, or using SELEX variants [6,7].

Another aspect to highlight is the preparation of aptamers, which typically consists of a very long protocol with various selection stages. Therefore, the use of technologies such as high-throughput sequencing, microfluidics, and microarrays has been proposed to reduce both costs and time associated with the preparation of aptamers. In addition, tools such as bioinformatics, machine learning, and artificial intelligence, will help to develop/identify new specific aptamers for analytes and predict in silico thermodynamic characteristics, affinity, and specificity. New technologies will optimize time and economic losses caused by 8–15 rounds of selection of aptamers using the SELEX method [6].

In summary, the properties of aptasensors or LOC aptasensors can be improved by using new nanomaterials, modifications in the nucleobases of aptamers, artificial DNA analogs, the use of multiple aptamers for a specific target, and the SELEX variants. In addition, the use of Bioinformatics, together with new technological trends, are tools with great potential that will allow the development of aptamers at low cost and reduced production times.

## 5. Conclusions

EPs have become a major environmental concern around the world. The environmental occurrence of EPs, such as pharmaceutical pollutants, EDCs, heavy metals, and agrochemicals, has been extensively documented, together with their negative effects on non-target organisms. Thus, the design and development of real-time and on-site detection systems are of interest to the scientific community to overcome the limitations presented by conventional analytical techniques. In this manner, aptamers have played a fundamental role as a recognition element in novel biosensors due to their sensitivity, selectivity, and flexibility, among other advantageous characteristics. In this review, we summarized and discussed the most recent advances in aptamer-based biosensors as a suitable alternative to sensitively detect pollutants of emerging concern. The limitations and current challenges faced by this technology were finally presented to propose future research directions to achieve practical applicability.

## Figures and Tables

**Table 1:** Summary of the application of aptasensors for the detection of EPs.

Types of Aptasensors	Pollutants Detected	LOD	Range of Detection	Sample Matrix	Reference
Photoelectrochemical	*E. coli* O15J_HJ	200 cfu/mL	4 × 10^2^ cfu/mL to 4 × 10^7^ cfu/mL	Water	[25]
Fluorescence	*Salmonella typhimurium*	1 cfu/mL	From 10 to 10^10^ cfu/mL	Water and food	[26]
Colorimetric	*Salmonella typhimurium*, *E. coli* and *Staphylococcus aureus*	≤10^4^ cfu/mL	NR	Food	[27]
Conductometric	SARS-CoV-2 and adenovirus	1 Pfu/mL for human adenovirus and 1 × 10^4^ copies/mL for SARS-CoV-2	From 6 pfu/mL to 6 × 10^4^ pfu/mL to adenovirus and 1 × 10^4^ to 1 × 10^8^ copies/mL for SARS-CoV-2	Water, saliva, and serum	[28]
Fluorescence	AMP and KAM	0.06 ng/L for ampicillin and 0.0150 ng/L for Kanamycin	From 0.5 to 500 ng/L for ampicillin and from 0.5 to 1000 ng/L for Kanamycin	Water	[29]
Colorimetric	OTC and KAM	1 ag/mL	From 10^−6^ to 10^5^ pg/mL	Food	[30]
SERS	DEHP	8 pM	From 0.008 to 182 nM	Food and water	[31]
Colorimetric	DEHP	1 ng/L	0.003–10 µg/L	Water	[32]
Fluorescence	Bisphenol	32 nM	0–1300 µM	Water	[33]
SERS	Bisphenol	0.75 pg/mL	From 0.001 to 100 ng/mL	Water	[34]
Electrochemical	Pb^2+^	0.096 µg/L	From 0.1 to 10 µg/L	Water and Soil	[35]
Electro-chemiluminescence	Pb^2+^	18.9 pM	50 pM 387.9 nM	Water	[36]
Colorimetric	As^3+^	1.97 ppm for soil samples and 14.44 ppb for aqueous samples	NR	Soil	[37]
Fluorescence	As^3+^	5 ng/L	From 10 ng/L to 10 mg/L	Water	[38]
Electrochemical	As^3+^	1.4 × 10^−7^ ng/mL	3.8 × 10^−7^−3.0 × 10^−4^ ng/mL	Water	[39]
Fluorescence	Cd^2+^	2.15 nM	From 7.19 nM to 5 µM	Water	[40]
Colorimetric	EDI and IBF	10 nM for edifenphos and 5 nM for Iprobenfos	From 5 to 25 nM for Edifenphos and from 10 to 100 nM for Iprobenfos	Food	[41]
Fluorescence	Organophosphorus	13.4 nM to 23.4 nM	NR	Food	[42]

Abbreviations: LOD, Limit of detection; NR, Not reported; Ampicillin, AMP; Kanamycin, KAM; Oxytetracycline, OTC; Phthalate, DEHP; As^3+^, (Arsenic III); Cd^2+^, (Cadmium II); Pb^2+^, (lead II); Edifenphos, EDI; Iprobenfos, IBF.
